# HIV-1 Specific Antibody Titers and Neutralization among Chronically Infected Patients on Long-Term Suppressive Antiretroviral Therapy (ART): A Cross-Sectional Study

**DOI:** 10.1371/journal.pone.0085371

**Published:** 2014-01-15

**Authors:** Johannes S. Gach, Chad J. Achenbach, Veronika Chromikova, Baiba Berzins, Nina Lambert, Gary Landucci, Donald N. Forthal, Christine Katlama, Barbara H. Jung, Robert L. Murphy

**Affiliations:** 1 Division of Gastroenterology, Northwestern University, Chicago, Illinois, United States of America; 2 Division of Infectious Diseases and Center for Global Health, Northwestern University, Chicago, Illinois, United States of America; 3 Division of Infectious Diseases, University of California Irvine, Irvine, California, United States of America; 4 Institute of Applied Microbiology, University of Natural Resources and Applied Life Sciences Vienna, Vienna, Austria; 5 Université Pierre et Marie Curie-Paris, Hôpital Pitié-Salpêtrière, Paris, France; Institute of Infection and Global Health, United Kingdom

## Abstract

The majority of potent and broadly neutralizing antibodies against HIV-1 have been isolated from untreated patients with acute or chronic infection. To assess the extent of HIV-1 specific antibody response and neutralization after many years of virologic suppression from potent combination ART, we examined antibody binding titers and neutralization of 51 patients with chronic HIV-1 infection on suppressive ART for at least three years. In this cross-sectional analysis, we found high antibody titers against gp120, gp41, and the membrane proximal external region (MPER) in 59%, 43%, and 27% of patients, respectively. We observed significantly higher endpoint binding titers for gp120 and gp41 for patients with >10 compared to ≤10 years of detectable HIV RNA. Additionally, we observed higher median gp120 and gp41 antibody titers in patients with HIV RNA <50 copies/mL for ≤5 years. 22% of patients neutralized a HIV-1 primary isolate (HIV-1_JR-FL_) and 8% neutralized a HIV-2/HIV-1 MPER chimera. Significantly greater HIV-1_JR-FL_ neutralization was found among patients with >10 years of detectable HIV RNA (8/20 [40.0%] versus 3/31 [9.7%] for ≤10 years, p = 0.02) and a trend toward greater neutralization in patients with ≤5 years of HIV RNA <50 copies/mL (7/20 [35.0%] versus 4/31 [12.9%] for >5 years, p = 0.08). All patients with neutralizing activity mediated successful phagocytosis of VLPs by THP-1 cells after antibody opsonization. Our findings of highly specific antibodies to several structural epitopes of HIV-1 with antibody effector functions and neutralizing activity after long-term suppressive ART, suggest continuous antigenic stimulation and evolution of HIV-specific antibody response occurs before and after suppression with ART. These patients, particularly those with slower HIV progression and more time with detectable viremia prior to initiation of suppressive ART, are a promising population to identify and further study functional antibodies against HIV-1.

## Introduction

A substantial amount of the antibody response in human immunodeficiency virus type 1 (HIV-1) infected individuals is directed against the envelope glycoprotein (Env) embedded on the viral surface [1]; however, only a minor fraction of these antibodies are able to recognize conserved epitopes on trimeric Env and thus elicit a consistent, broad, and potent neutralization of HIV-1 [2], [3]. Distinguished epitopes prone to cross-neutralization include but are not limited to the membrane proximal external region (MPER) on gp41 [4], [5], the CD4 binding site (CD4bs) [6], [7], glycan based epitopes [8], variable loops 1 and 2 (V1/V2) [9], and the variable loop 3 (V3) region [10] on gp120.

The majority of potent and broadly neutralizing HIV-1 monoclonal antibodies (mAbs) targeting these conserved regions were isolated from individuals with untreated acute or advanced chronic HIV infection when HIV RNA levels are highest [11]. Additionally, increased breadth and potency of isolated neutralizing antibodies were associated with low CD4+ T cell counts and high HIV RNA levels [3], [12], [13]. The direct correlation between high HIV RNA level and greater neutralization of HIV-1 specific antibodies was also observed among elite HIV controllers or suppressors (ES) not on antiretroviral therapy (ART) [14]. Doria-Rose and colleagues found that elite suppressors (with undetectable HIV RNA off ART) were less likely to generate broadly neutralizing antibodies than progressors or long term non-progressors with detectable HIV viremia [15]. Therefore, HIV-infected individuals with suppressed viremia (with or without ART) were considered poor candidates to evaluate for broadly neutralizing HIV-1 specific antibodies to novel epitopes [16].

HIV-1 envelope specific titers and neutralization clearly decrease after initiation of suppressive ART during acute infection [17]–[19]. However, a recent study reported high antibody titers with modest neutralization when ART was initiated several years after established chronic infection [20]; thus, raising the possibility that HIV-1 specific immune responses evolve over time on ART. Additionally, it has been found that on suppressive ART, B cell counts increase, B cell subpopulations normalize, and B cell activation persists [21], [22]. Recent evidence suggests that compartmentalized HIV replication and very low-level HIV viremia persist on suppressive ART [23]–[25]. We hypothesized that functional B cells responding to HIV antigen in lymphatic tissues, in the setting of immune recovery on ART, evolve a more effective humoral immune response. To improve our understanding of this type of autologous antibody response, we examined HIV-specific antibodies, neutralization, and effector functions among a population of patients on long-term suppressive ART with immune recovery. Although there is clear evidence from numerous non-human primate studies that neutralizing antibodies can prevent HIV-1 acquisition [26]–[31] little is known about their role in preventing or controlling established infection in humans [1], [32]–[34]. Therefore, it is important to further the knowledge of humoral immunity in HIV-1 infected patients (with and without ART) and study the role of HIV-1 specific antibodies and their putative effector functions on virus transmission and pathogenesis.

## Materials and Methods

### Antibodies, viruses and peptides

Michael B. Zwick and Dennis R. Burton kindly provided mAbs Z13e1 [35] and b12 [36]. MAbs 1F7, 2G12, 4E10, and 2F5 were generously donated from Dietmar Katinger (Polymun Scientific) and Hermann Katinger [37]. MAb 17b was obtained through the IAVI NAC reagent repository after generous donation from James Robinson [38]. HIV-2/HIV-1 MPER chimeras were kindly provided by George M. Shaw [39]. MPER peptides were generously provided by Sampat Ingale and Philip Dawson [40].

### Study population, serum samples, data collection, and ethics statement

We obtained sera from 51 adult patients consented and evaluated for participation in the Eramune 02 clinical trial (http://clinicaltrials.gov/ct2/show/NCT00976404) at the Northwestern University clinical site in Chicago, USA. Patients were assessed if they had current CD4+ cell counts ≥350 cells/µl, continuous suppression of HIV RNA <50 copies/mL for at least one year and <500 copies/mL for at least three years. We excluded patients from evaluation who had any of the following conditions: diagnosis of cancer within the last five years, history of autoimmune disease, immunologic therapeutic intervention within the past year, and any co-morbid condition with an expected survival of less than twelve months. As per Eramune 02 evaluation procedures, serum and cell pellets were obtained for evaluation of adenovirus antibodies and total proviral HIV DNA assessment. Clinical information including demographics, HIV diagnosis dates, current ART regimens, CD4+ cell counts, CD8+ cell counts, nadir CD4+ cell counts, HIV RNA levels, and dates of HIV suppression were collected by electronic chart review and patient interview.

All patients included in this study provided written informed consent. The ERAMUNE 02 protocol and informed consent form were approved by the Northwestern University Institutional Review Board. Laboratory specimens were completely anonymized and unlinked from any patient identifying information.

### Enzyme linked immunosorbent assay (ELISA)

Patient sera were screened for specific antibody titers against recombinant gp120_JR-FL_, gp41_JR-FL_ [35], and p24. To test whether any gp41 specific antibodies were directed against the MPER, antibody binding was assessed against three MPER mimetics: QIQQEKNMYELLALDKWASLWNWFDITKWLWYIKYGVYIV – designated as MPER-p1 according to the MPER sequence of the HIV-2/HIV-1 Env chimera 7312-C1 – [41], LLELDKWASLWNWFDITNWLWYIKKKK – designated as MPER-p2, and NWFDITNWLWYIKKKK-NH2 – designated, as MPER-p3 – [35], [42].

To further examine the antibody response against gp120, patient sera were evaluated for binding against recombinant gp120_JR-FL_ mutants lacking either the V1/V2 loop (ΔV1/V2) or the V3 loop (ΔV3). Specific binding of human mAbs 2G12, 1F7, 2F5, Z13e1, 4E10, and a human serum control to the above-described antigens was determined in parallel. Direct binding ELISA was performed according to Gach and colleagues [43]. In brief, 96 well plates were coated with 1µg/mL of antigen (50 ng/well) over night a 4°C. Plates were washed, blocked and incubated with serially diluted serum samples and antibody controls for 1 hour at room temperature. Bound antibodies were detected with a HRP labeled goat anti human Fc antibody (Jackson ImmunoResearch) developed with TMB substrate and read with a BIOTEC plate reader.

### Soluble CD4 (sCD4) inhibition assay

Sera from patients #12, #37, and #42 who had no significant decline in V1/V2 and V3 loop mutant binding were tested for the presence of CD4bs specific antibodies. For this approach, serially diluted sera and antibody controls were incubated with a constant concentration of sCD4 (10 µg/mL) to compete for binding with recombinant gp120_JR-FL_ as described previously [43].

### Serum antibody purification

HIV-1 patient serum samples were purified using 0.2 mL NAb protein A/G spin columns (Thermo Scientific) according to the manufacturer's instructions. Fractions containing the purified antibodies were further concentrated and buffer exchanged using Amicon Ultra 0.5 mL centrifugal filter (50 KDa) units (Millipore). IgG concentration was measured at OD_280_ with a NanoDrop Spectrophotometer (Thermo Scientific).

### Virus production and neutralization assay

Pseudotyped viruses (HIV-1_JR-FL_, VSV-g pseudotyped HIV-1) were produced as described recently [44]. Replication competent viruses (HIV-2/HIV-1 chimeras) were generated identically but in the absence of the HIV-1 backbone plasmid pSG3ΔEnv. To evaluate patient samples for their neutralization potency, serum was heat inactivated and 0.2 µm filtered before virus incubation. In a preliminary neutralization experiment, crude serum samples were tested against pseudotyped HIV-1_JR-FL_. Over 65% of the sera were able to neutralize HIV-1_JR-FL_ with a reciprocal serum dilution factor ranging from 100 up to >2700 (data not shown). To minimize false positive results due to the presence of antiretroviral drugs (e.g. efavirenz) in serum, IgG was purified prior to testing. None of the purified patient samples neutralized a VSV-g pseudotyped HIV-1 virus control confirming successful removal of antiretroviral drugs during IgG purification [20]. In the next step, crude sera were screened for MPER neutralizing antibodies, as the antiretroviral drugs did not affect neutralization against HIV-2 chimeras. Single round infectivity assays were performed and evaluated as described by Walker and colleagues [45].

### Antibody effector function assay

To test for antibody effector functions of neutralization positive patients, purified IgG fractions were serially diluted in the presence of green fluorescence protein labeled Env expressing virus like particles (VLPs). VLPs were opsonized for 1 hour at 37°C followed by a 1 hour incubation step with Thp-1 cells. Phagocytosis of VLPs was determined by flow cytometry as described previously [46].

### Total proviral HIV DNA assessment

As part of the evaluation procedures for Eramune 02, we isolated PBMC pellets from each patient and performed a real time PCR assay for the semi-quantitative detection of HIV-1 cellular DNA. The methodology of this research assay has been described elsewhere [47].

### Statistical analysis

We performed bivariate comparisons of log_10_-transformed serum antibody endpoint titers for demographic, clinical, and treatment characteristics using Fisher's exact or Mann Whitney Wilcoxon rank sum tests for dichotomous or continuous variables, respectively. As estimates of extent of HIV disease, burden of latent HIV reservoir, and replication, we compared mean HIV specific antibody titers for the following variables: a) nadir CD4+ cell count categorized as ≤200 versus >200 cells/µl, b) total proviral HIV DNA categorized as ≤150 and >150 copies/10^6^ PBMCs, c) time with detectable HIV RNA categorized as ≤10 and >10 years, and d) time with HIV RNA <50 copies/mL categorized as ≤5 and >5 years. We also evaluated correlations between log_10_-transformed serum antibody titers and age, nadir CD4+ cell count, current CD4+ cell count, current CD8+ cell count, current CD4+/CD8+ ratio, current total proviral HIV-1 DNA level, time with detectable HIV RNA, time with HIV RNA <50 copies/mL, and adenovirus antibody titer using Pearson's correlation. We performed multivariable analyses of antibody neutralization controlling for age, race, sex, and nadir CD4+ cell count using logistic regression. All descriptive, comparative and correlative statistics were performed using SAS 9.3 or Graph Pad Prism 6.0. Test results were considered statistically significant for two-sided p-value <0.05.

## Results

### Study population

A total of 51 patients were evaluated for participation in the Eramune 02 clinical trial and included in this study (Table 1). The median age was 50 years (interquartile range [IQR] 46 – 55), 45/51 (88%) were male and 36/51 (71%) white race. The majority of patients were on non-nucleoside reverse transcriptase inhibitor (NNRTI) based ART (29/51 [57%]) and 27/51 (53%) had ART regimens containing efavirenz. The median time since HIV diagnosis was 13.4 years (IQR 9.3 – 19.6), median time with detectable HIV RNA was 7.1 years (IQR 3.5 – 13.2), and median time with HIV RNA <50 copies/mL was 5.4 years (IQR 4.4 – 6.2). The median total proviral HIV-1 DNA was 159 copies/10^6^ PBMCs (IQR 45 – 425), median nadir CD4+ cell count was 217 cells/µl (IQR 116 – 435), median current CD4+ cell count was 613 cells/µl (IQR 508 – 797), median current CD8+ cell count was 728 (IQR 509 – 1045) and median current CD4+/CD8+ ratio was 0.83 (IQR 0.58 – 1.24).

**Table 1:** Patient characteristics and results of antibody assays.

Characteristic or Result (total n = 51)	
**Age, median years (IQR)**	50 (46,55)
**Male sex (%)**	45 (88)
**White race (%)**	36 (71)
**Type of ART**	
** Boosted PI (%)**	18 (35)
** NNRTI (%)**	29 (57)
** Boosted PI+NNRTI (%)**	2 (4)
**Efavirenz in ART regimen**	27 (53)
**Nadir CD4+ count, median cells/µL (IQR)**	217 (116,435)
**Current CD4+ count, median cells/µL (IQR)**	613 (508,797)
**Current CD8+ count, median cells/µL (IQR)**	728 (509,1045)
**Current CD4+/CD8+ Ratio, median (IQR)**	0.83 (IQR 0.58,1.24)
**Total Proviral HIV DNA, median copies/10^6^ PBMCs (IQR)**	159 (45,425)
**Total time from HIV diagnosis, median years (IQR)**	13.4 (9.3,19.6)
**Time with detectable HIV RNA, median years (IQR)**	7.1 (IQR 3.5,13.2)
**Time with HIV RNA <50 copies/mL, median years (IQR)**	5.4 (4.4,6.2)
**Adenovirus antibody, median titer (IQR)**	43 (12,3704)
**Antibody Epitope, median endpoint titer log_10_ (IQR)**	
** p24**	5.2 (4.6,5.7)
** gp120**	6.1 (5.5,6.3)
** gp41**	6.0 (5.4,6.2)
** MPER-p1**	3.8 (3.3,4.1)
** MPER-p2**	3.4 (3.3,3.8)
** MPER-p3**	3.8 (3.4,4.0)
**HIV-1_JR-FL_ Neutralization**	11 (22)

### Overall antibody titers

**Figure 1:**
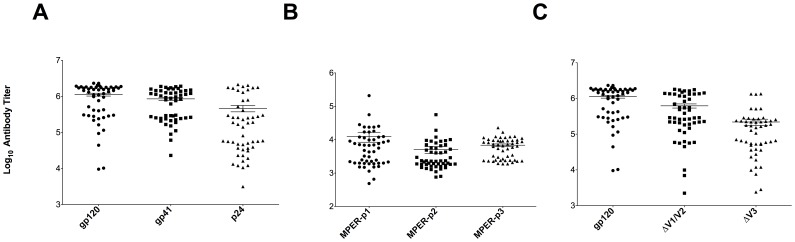
Serum mapping against different HIV-1 epitopes. Sera of HIV-1 patients were tested for binding against different HIV-1 antigens including recombinant gp120_JR-FL_, gp41_JR-FL_, and p24 (A). To check for MPER specific antibodies, serum binding against three different MPER peptides was evaluated (B). To gain better knowledge about the binding epitopes on gp120, sera were tested against the gp120 variable loop truncation variants ΔV1/V2 and ΔV3 (C). Endpoint titers of the patient samples were determined by calculating the highest serum dilution that gives a reading above the blank including three standard deviations.

Over half of the patients (59%) revealed antibody titers equal or higher than 1×10^6^ (high) against recombinant gp120_JR-FL_. Binding titers between 1×10^5^ and 1×10^6^ (medium) against gp120_JR-FL_ were observed in 33% of the patients whereas only 8% exhibited antibody titers below 1×10^5^ (low) (Figure 1A). A similar distribution was seen for recombinant gp41_JR-FL_ binding, where 43% of the patients revealed high binding titers greater than 1×10^6^ followed by 51% with medium binding titers, and 6% with binding titers below 1×10^5^ (Figure 1A). One fifth of the patients showed high titers against p24, followed by 33% with medium binding titers against the HIV-1 capsid protein (Figure 1A).

### Antibodies to MPER and gp120 variable loops

Patient sera exhibited notable MPER specific antibody responses ranging from 1×10^4^ to 5.5×10^4^. MPER-p1 was recognized by 27% of the patients at an antibody titer >1×10^4^, followed by MPER-p3 with 20%, and MPER-p2 with 8%. Patient #26, revealed an exceptional antibody titer (>2×10^5^) against MPER-p1 (Figure 1B). 55% of the patients showed a ≥50% decrease (ranging from 2-fold to 7-fold) in antibody titers against the ΔV1/V2 loop mutant compared to gp120 wild type (WT). In case of the second gp120 mutant, ΔV3, the effects were even more pronounced since 94% of the patients revealed a reduction (≥50%) in antibody binding compared to the WT with a median drop in antibody levels of 7-fold (Figure 1C). These results confirm the presence of V1/V2 loop specific antibodies and, to a higher extent, V3 loop specific antibodies in the sera. In contrast, three of the patient serum samples (#12, #37, and #42) exhibited distinct antibody populations, which were not affected by the gp120 loop deletions (Figure 1C). Binding of antibody and serum controls against each above described antigen is summarized in Figure S1.

### Antibodies to CD4bs

**Figure 2:**
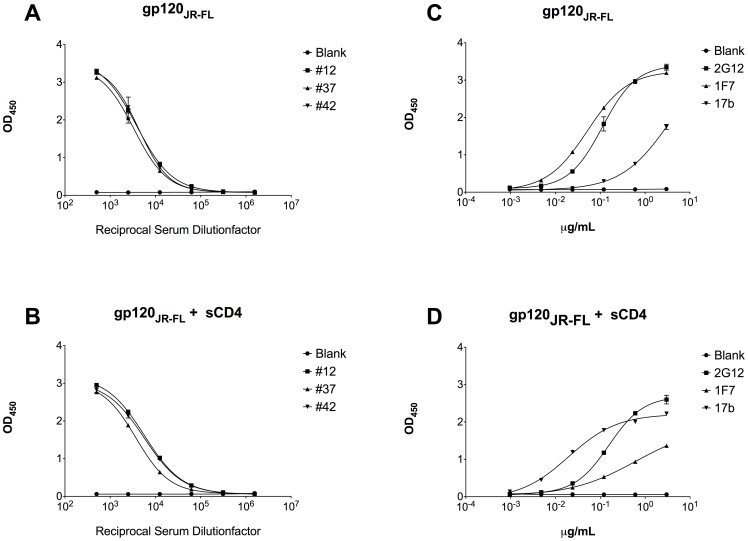
Soluble CD4 competition assay. Assay was performed with three HIV-1 positive serum samples and respective mAb controls (1F7, 2G12, and 17b) in the absence (A and C) or presence (B and D) of soluble CD4. The EC_50_ values (half maximal binding at reciprocal serum dilution or antibody concentration) were calculated and compared for significant differences.

The presence of sCD4 did only marginally alter gp120 binding for patients #12, #37, and #42 (Figure 2A and 2B). We observed 1.5-fold (#12), 1.1-fold (#37), and 1.3-fold (#42) higher EC_50_ values in the presence of sCD4 suggesting occurrence of CD4bs antibodies in these patients although at a low quantity (Table S1). Antibody control 2G12 revealed an EC_50_ of 0.156 µg/mL in the presence of sCD4 compared to an EC_50_ of 0.112 µg/mL in the absence of sCD4 (Figure 2C and D). On the contrary, mAb 1F7, a CD4bs specific antibody [43], [48], exhibited a 12-fold higher EC_50_ value in the presence of sCD4 (EC_50_ = 0.052 µg/mL) than without (EC_50_ = 0.624 µg/mL), indicating sCD4 competition. Binding enhancement was observed for mAb 17b (EC_50_ = 3.726 µg/mL) where the presence of sCD4 decreased the EC_50_ value more than 186-fold (EC_50_ = 0.02 µg/mL).

### Antibody neutralization and effector functions

22% of the patients were able to neutralize HIV-1_JR-FL_ pseudotyped virions after IgG purification with a median IC_50_ of 42.9 µg/mL (Table 2). Patient #48 exhibited a 14-fold lower IC_50_ value than the median IC_50_ value of all neutralizing patient samples. As shown in Table 2, four patients (7.8%) were able to neutralize MPER chimera 7312-C1 (subtype B HIV-1 MPER) with a serum dilution ranging from 1∶108 to 1∶125. No sign of neutralization was observed with the MPER clade C consensus variant 7312-C1C or the HIV-2 WT 7312A. Neutralization values of human mAb controls at a starting concentration of 5 µg/mL are shown in Table S2. Since patient #48 showed notable neutralization potency against HIV-1_JR-FL_ the sample was further tested against a small panel of different HIV-1 clades (i.e. AE, B, C, and E) for potential cross-neutralization breadth (Table 3). Human mAbs b12 and 2G12 were included as a control at a starting concentration of 5 µg/mL. Patient #48 was able to neutralize eight out of twelve viruses including five viruses from clade B (HIV-1_MN_, HIV-1_TRO.11_, HIV-1_JR-CSF_, HIV-1_SF-162_, and HIV-1_JR-FL_), two clade C viruses (HIV-1_ZM53M_, HIV-1_CAP045_), and one clade E virus (HIV-1_93TH966.8_). A similar pattern was seen for mAb b12, which was able to neutralize six viruses at a concentration below 5 µg/mL. However, b12 was not able to neutralize HIV-1_TRO.11_ and HIV-1_93TH966.8_ (clade B and clade E, respectively). Antibody 2G12 neutralized a total of five clade B viruses and none of the other clades tested at the highest concentration. None of the antibodies were able to neutralize the VSV-g pseudotyped HIV-1 virus control (data not shown).

**Table 2:** Summary of patients with neutralizing activity against HIV-1_JR-FL_ and/or HIV-2/HIV-1 7312-C1.

Patient ID #	IC_50_ * (μg/mL)		IC_50_?		
	Virus		Virus		
	JR-FL	VSV-g	7312-C1	7312-C1C	7312A
9	34.1	> 200	< 100	< 100	< 100
10	56.0	> 200	< 100	< 100	< 100
11	125.0	> 200	< 100	< 100	< 100
12	42.9	> 200	108	< 100	< 100
18	127.3	> 200	116	< 100	< 100
26	> 200	> 200	109	< 100	< 100
32	65.5	> 200	< 100	< 100	< 100
35	41.5	> 200	< 100	< 100	< 100
41	38.7	> 200	< 100	< 100	< 100
42	17.5	> 200	< 100	< 100	< 100
48	3.0	> 200	125	< 100	< 100
50	77.8	> 200	< 100	< 100	< 100

Polyclonal inhibitory concentration of purified IgG fractions

^?^ Reciprocal dilution factor of serum samples

**Table 3:** Neutralization potency and breadth of HIV-1 patient #48 against a small panel of different HIV-1 viruses including clades AE, B, C, and E.

Clade	Virus	Patient #48	b12	2G12
		IC_50_ * (μg/mL)	IC_50_ (μg/mL)	
**AE**	C1080	> 200	> 5	> 5
**B**	MN	< 1.0	< 0.02	> 5
**B**	TRO.11	9.5	> 5	0.19
**B**	JR-CSF	12.9	0.26	0.4
**B**	SF162	< 1.0	0.02	0.14
**B**	JR-FL	3.4	0.03	1.1
**B**	PVO.4	> 200	> 5	0.41
**C**	DU156.12	> 200	> 5	> 5
**C**	93MW959	> 200	> 5	> 5
**C**	ZM53M	5.7	0.13	> 5
**C**	CAP045	6.0	1.1	> 5
**E**	93TH966.8	4.6	> 5	> 5

Polyclonal inhibitory concentration of purified IgG fractions

**Figure 3:**
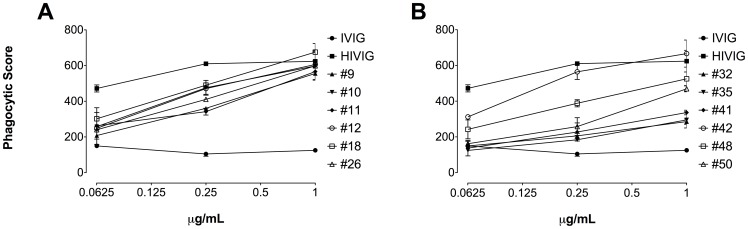
Phagocytosis assay. Phagocytic score of samples tested at different antibody concentrations is indicated. All purified IgG fractions and the positive control HIVIG revealed Fc-mediated effector functions compared to the negative control IVIG. The cut off value of confidence interval for IVIG has a phagocytic score of 194.77. All samples greater than the cut off are positive with 99% confidence.

Next, we looked at antibody effector functions of purified neutralizing IgG fractions against green fluorescence protein labeled Env expressing VLPs. VLPs were phagocytosed very efficiently by all patient samples compared to the negative control IVIG. Patients #42, #18, and #10 revealed the highest phagocytosis levels starting at a concentration of 1.0 µg/mL. HIVIG was used as a positive control for successful phagocytosis of VLPs (Figure 3).

### Patient characteristics and antibody comparisons

There were no statistically significant differences in any HIV-1 specific antibody endpoint binding titers for patients with nadir CD4+ cell count ≤200 cells/µl compared to those with >200 cells/µl for any of the epitopes analyzed. Patients with nadir CD4+ cell count >200 cells/µl experienced non-significantly greater HIV-1_JR-FL_ neutralization with 8/29 [27.6%] compared to 3/22 [13.6%] for those with CD4+ cell counts ≤200 cells/µl exhibiting any activity, p = 0.31. In terms of total HIV-1 proviral DNA, there were also no statistically significant differences in any of the HIV-1 specific antibody endpoint binding titers between patients with ≤150 and >150 copies/10^6^ PBMCs. Patients with total HIV-1 proviral DNA >150 copies/10^6^ PBMCs tended to have greater neutralization with 8/26 [30.8%], compared to 3/25 [12.0%] for those with ≤150 copies/10^6^ PBMCs, exhibiting any activity against HIV-1_JR-FL_ pseudotyped virus (p = 0.17).

**Figure 4:**
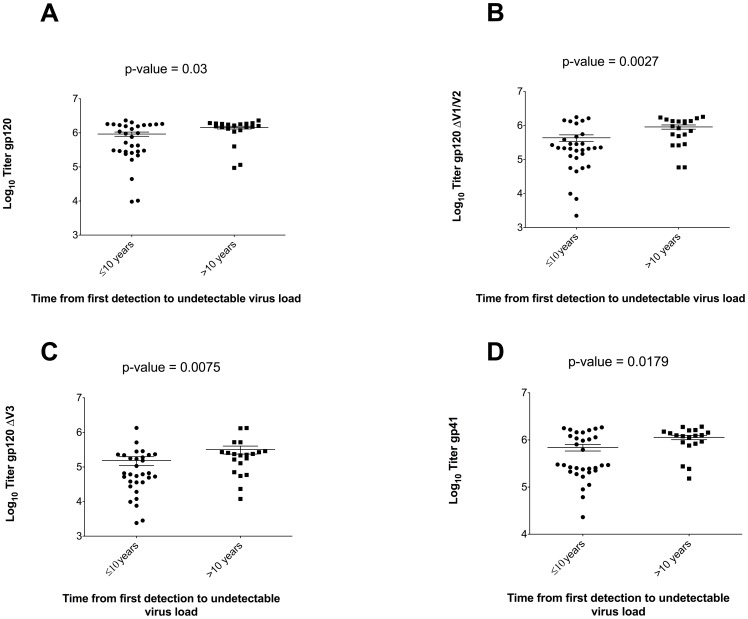
Comparison of HIV-1 specific antibody endpoint titers and time with detectable HIV RNA categorized as ≤10 and >10 years. Scatter plots of antibody endpoint titers to recombinant gp120_JR-FL_, gp120_JR-FL_ ΔV1/V2, gp120_JR-FL_ ΔV3, and gp41 categorized by time with detectable HIV RNA show significantly higher titers for patients with >10 years of detectable viremia.

**Figure 5:**
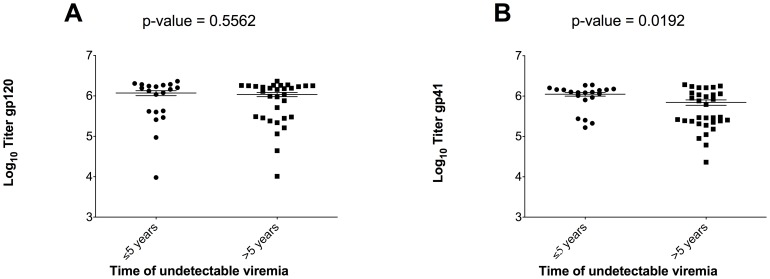
Comparison of HIV-1 specific antibody endpoint titers and time with HIV RNA<50 copies/mL categorized as ≤5 and >5 years. Median binding titers against gp120_JR-FL_ (A) and gp41_JR-FL_ (B) were both lower in patients with >5 years of undetectable viremia.

We did find significantly higher endpoint binding titers for gp120_JR-FL_ (p = 0.03), gp120_JR-FL_ ΔV1/V2 (p = 0.003), gp120_JR-FL_ ΔV3 (p = 0.01) and gp41_JR-FL_ (p = 0.02) for patients with >10 compared to ≤10 years of detectable HIV RNA (Figure 4A to D). There were no statistically significant differences in HIV-1 specific antibody endpoint titers for patients with ≤5 compared to >5 years with HIV RNA <50 copies/mL, except gp41, for which we observed a higher median antibody titer in patients with HIV RNA <50 copies/mL for ≤5 years (6.1 versus 5.5 log_10_, p = 0.02). A similar trend, although not statistically significant (p = 0.56), was observed for gp120 (Figure 5). We found significantly greater HIV-1_JR-FL_ neutralization among patients with >10 years of detectable HIV RNA (8/20 [40.0%] versus 3/31 [9.7%] for ≤10 years, p = 0.02) and a trend toward greater neutralization in patients with ≤5 years of HIV RNA <50 copies/mL (7/20 [35.0%] versus 4/31 [12.9%] for >5 years, p = 0.08). In multivariable analyses controlling for age, race, sex, and nadir CD4+ cell count, the odds of HIV-1_JR-FL_ neutralization were significantly greater for patients with >10 years of detectable HIV RNA (adjusted OR = 7.7; 95% CI: 1.4-43.3, p = 0.02) before ART initiation, and again a trend toward greater neutralization among those with ≤5 years of HIV RNA <50 copies/mL (adjusted OR = 4.9; 95% CI: 0.95–25.6, p = 0.06).

Additionally, we found direct correlations between certain patient characteristics and HIV-1 specific antibody endpoint titers or neutralization levels as follows: age and MPER-p2 (r = 0.30, p = 0.03); current CD4+ cell count and MPER-p2 (r = 0.22, p = 0.12); time with detectable HIV RNA and gp120 (r = 0.28, p = 0.04), gp41 (r = 0.27, p = 0.05), and HIV-1_JR-FL_ neutralization (r = 0.40, p = 0.004); total proviral HIV-1 DNA and MPER-p1 (r = 0.30, p = 0.04) and MPER-p2 (r = 0.31, p = 0.03).

## Discussion

The recent discovery of potent and broadly neutralizing antibodies to HIV-1 from infected individuals has galvanized interest in protective or therapeutic interventions harnessing the humoral immune response [49]. Using the latest techniques, investigators have largely focused on natural production of these antibodies in acute or early untreated HIV infection where they arise in approximately 20% of infected individuals [50]. Several longitudinal studies of patients treated with ART during acute HIV-infection showed a decline in HIV-1 specific antibody levels over time. This was thought to be due to the rapid control of viremia and reduced time of antigen stimulation [17], [51] as well as reduced B cell hyperresponsiveness [52]. In contrast, when ART was initiated several years after established chronic infection, there is persistence of antibody production against HIV-1 structural proteins [18], [20] or in some cases even augmentation of antibody titers after ART treatment [53]. In our study, we observed exceptionally high antibody titers against recombinant gp120_JR-FL_, and gp41_JR-FL_ in the majority of patients on long-term suppressive ART. We found that antibody titers against gp120 and gp41 were significantly higher for patients who had longer time with detectable viremia (>10 years). Additionally, we found a trend toward lower median gp120 and gp41 antibody levels in patients who had greater time with undetectable viremia (>5 years). Our findings confirm the association between viremia and HIV specific antibodies, but further illustrate the slow clearance of HIV-specific antibodies over longer times on suppressive ART. This could be due to continuous antigenic stimulation or persistent immune dysfunction and antibody production even when virus is controlled with ART [25], [53].

Antibodies against gp41 are the first line of defense in acute HIV-1 infection [54]; however, the majority of gp41-binding antibodies produced in chronic HIV-1 infection are generated from memory B cells previously activated by non–HIV-1 antigens and thus unable to elicit a neutralizing immune response [55]. The MPER is one of the best-characterized regions on gp41 since it is the target of four broad and potent neutralizing antibodies including 2F5, 4E10, Z13e1, and 10E8 [56]–[59]. Moreover the MPER is highly conserved across clades and thus represents a promising vaccine target [4], [60]. To investigate whether patients on long-term suppressive ART develop highly specific antibodies against the MPER, we tested their sera against three MPER mimetics. We found notable antibody titers (>1×10^4^) against all peptides. Our titers were consistent with previously described MPER antibody titers from other individuals with chronic HIV-infection [41], [61], but our patients were unique in that they all had achieved long-term virologic suppression on ART. Interestingly, patient #26 exhibited exceptionally high titers against MPER-p1 although we believe that most of the antibody response was directed against the N-terminus of the peptide as antibody titers against an overlapping MPER-p2 peptide were almost 30-fold lower. We also found that height of MPER binding titers did not necessarily correlate with neutralization. For example, patient #6 revealed an MPER specific antibody titer of 5.5×10^4^ against both MPER-p1 and MPER-p2 but did not show any neutralization activity against HIV-2/HIV-1 7312-C1. One possible explanation for this is that these antibodies may recognize a linear, instead of a conformational, epitope. On the contrary, patient #48 exhibited the greatest neutralization (1∶125) although the MPER specific titer was marginally lower than 1×10^4^. We also observed overall higher titers against MPER-p3 compared to MPER-p2, which overlap in 13 MPER specific amino acids. We speculate the difference was due to enhanced exposure of accessible sites on the shorter peptide or avidity effects whereby the antibody interacts with two peptides. All four patients with neutralizing MPER antibodies exhibited binding titers close to 1×10^4^ or greater than 1×10^4^ against MPER-p1. Identification of binding titers to this peptide could be a simple and valuable screening tool in future studies exploring neutralizing MPER antibodies.

Within the trimeric envelope spike, variable gp120 loops V1/V2 and V3 play dual roles in antibody recognition. They contain epitopes prone to neutralization and block key neutralizing epitopes on the CD4bs and sites of conformational change after CD4 binding [62], [63]. The importance of these V1/V2 and V3 regions for the generation of broadly neutralizing antibodies has been investigated by several studies [64]-[66]. Our gp120 epitope analyses revealed a higher proportion of V3 loop (48/51; 94%) compared to V1/V2 loop (28/51, 55%) specific antibodies. Additionally, we observed a higher frequency of V1/V2/V3 specific antibodies among patients with less than 10 years of detectable viremia. This is in agreement with previous reports where early autologous neutralizing antibody responses predominantly target variable epitopes that are exposed on Env including V1/V2 and V3 [67], [68]. Antibodies against V3 usually develop faster and reach higher titers compared to antibodies against other highly conserved epitopes such as CD4bs or MPER [69].

Interestingly, three of the patients showed an average one-fold reduction in binding against V1/V2 and 1.4-fold reduction in V3; however, when we checked for CD4bs antibodies, we observed only a marginal decrease in binding (1.5-fold to 1.1-fold) in the presence of sCD4. Nevertheless, two of these patients (#12 and #42) were able to neutralize HIV-1_JR-FL_ in a single round infectivity assay suggesting either the occurrence of potent CD4bs-specific and V1/V2/V3-specific antibodies or the existence of neutralizing antibodies to other epitopes such as glycans [70], [71].

We found a total of 12 patients (23.5%) with neutralization activity against HIV-1_JR-FL_, HIV-2/HIV-1 7312 C1 or both. One patient had neutralization activity across both viruses (1.9%). Our neutralization results were similar to a recent study where 1.7% of patients on suppressive ART were able to neutralize a similar panel of viruses [20]. We observed significantly greater neutralization in patients with more time with detectable HIV viremia and a trend toward greater neutralization with higher nadir CD4+ cell counts and higher current total proviral HIV-1 DNA. Again, this illustrates an evolving partially effective antibody response as HIV disease advances with uncontrolled viremia. Alternatively, these patients were able to naturally control HIV progression and avoid initiation of ART for longer periods of time due to an enhanced antibody response. Given the cross-sectional nature of our study, we are unable to fully understand the relationship between antibody neutralization and HIV replication over time.

Other mechanisms distinguished from antibody neutralization by Fab are Fc-mediated effector functions, such as phagocytosis and lysis, which also play an important role in the humoral immune response to HIV infection [72], [73]. Essentially, the Fcγ portion of anti-HIV antibodies interact with the corresponding Fcγ receptor I or II on the cell surface of antigen presenting cells, thereby mediating HIV inhibition by a mechanism involving the phagocytosis and clearance of HIV-IgG immune complexes [46]. In our study, we found a positive correlation between antibody titers and phagocytosis levels. Patients #42, #18, and #10 showed the highest antibody titers against gp120 and gp41 along with the highest phagocytosis levels. Thus, our findings confirm that there are antibody-mediated effector functions present in addition to those leading to HIV neutralization.

In summary, our findings of high antibody binding titers to several structural epitopes of HIV-1 with antibody effector functions and neutralizing activity after long-term suppressive ART, suggest antigenic stimulation and antibody production continues for many years after systemic HIV RNA levels reach undetectable. Moving forward, the longitudinal antibody response before and after suppressive ART requires further research but we believe our study is an encouraging first step that warrants future investigation of key patient populations. Furthermore, screening for autologous broadly neutralizing antibodies in this population should be targeted at those with several years of detectable viremia prior to initiation of suppressive ART.

## Supporting Information

Figure S1 **Monoclonal antibody and serum control binding titers against various antigens.** (A), gp120_JR-FL_ wild type (B), gp120_JR-FL_ V1/V2 loop deletion variant C), and gp120_JR-FL_ V3 loop deletion variant (D). 2G12 and 1F7 only recognized gp120_JR-FL_, whereas 2F5, Z13e1, and 4E10 interacted with gp41 and MPER. MPER-p2 was only recognized by mAb 4E10 since the epitopes of 2F5 and Z13e1 are missing No significant binding was detected against p24. The human serum control revealed only minor binding against all tested antigens.
(TIF)

Table S1 **EC_50_ values of three potential CD4 binding site antibody- containing serum samples.**
(DOCX)

Table S2 **Neutralization assay with HIV-1_JR-FL_ and HIV-2/HIV-1 MPER chimeras using monoclonal antibody controls.**
(DOCX)

## References

[1] Overbaugh J, Morris L (2012) The Antibody Response against HIV-1. Cold Spring Harb Perspect Med 2: a007039. 22315717 10.1101/cshperspect.a007039 PMC3253031

[2] Tomaras GD, Yates NL, Liu P, Qin L, Fouda GG, et al (2008) Initial B-cell responses to transmitted human immunodeficiency virus type 1: virion-binding immunoglobulin M (IgM) and IgG antibodies followed by plasma anti-gp41 antibodies with ineffective control of initial viremia. J Virol 82: 12449–12463. 18842730 10.1128/JVI.01708-08 PMC2593361

[3] Mikell I, Sather DN, Kalams SA, Altfeld M, Alter G, et al (2011) Characteristics of the earliest cross-neutralizing antibody response to HIV-1. PLoS Pathog 7: e1001251. 21249232 10.1371/journal.ppat.1001251 PMC3020924

[4] Gach JS, Leaman DP, Zwick MB (2011) Targeting HIV-1 gp41 in close proximity to the membrane using antibody and other molecules. Curr Top Med Chem 11: 2997–3021. 22044228 10.2174/156802611798808505

[5] Montero M, Gulzar N, Klaric KA, Donald JE, Lepik C, et al (2012) Neutralizing epitopes in the membrane-proximal external region of HIV-1 gp41 are influenced by the transmembrane domain and the plasma membrane. J Virol 86: 2930–2941. 22238313 10.1128/JVI.06349-11 PMC3302331

[6] Wyatt R, Kwong PD, Desjardins E, Sweet RW, Robinson J, et al (1998) The antigenic structure of the HIV gp120 envelope glycoprotein. Nature 393: 705–711. 9641684 10.1038/31514

[7] Lynch RM, Tran L, Louder MK, Schmidt SD, Cohen M, et al (2012) The development of CD4 binding site antibodies during HIV-1 infection. J Virol 86: 7588–7595. 22573869 10.1128/JVI.00734-12 PMC3416294

[8] Langedijk JP, Schuitemaker H (2012) A sweet surprise for HIV broadly neutralizing antibodies. Nat Med 18: 1616–1617. 23135511 10.1038/nm.2993

[9] Pinter A, Honnen WJ, Kayman SC, Trochev O, Wu Z (1998) Potent neutralization of primary HIV-1 isolates by antibodies directed against epitopes present in the V1/V2 domain of HIV-1 gp120. Vaccine 16: 1803–1811. 9795384 10.1016/s0264-410x(98)00182-0

[10] Zolla-Pazner S (2005) Improving on nature: focusing the immune response on the V3 loop. Hum Antibodies 14: 69–72. 16720976

[11] van Gils MJ, Sanders RW (2013) Broadly neutralizing antibodies against HIV-1: templates for a vaccine. Virology 435: 46–56. 23217615 10.1016/j.virol.2012.10.004

[12] van Gils MJ, Euler Z, Schweighardt B, Wrin T, Schuitemaker H (2009) Prevalence of cross-reactive HIV-1-neutralizing activity in HIV-1-infected patients with rapid or slow disease progression. AIDS 23: 2405–2414. 19770692 10.1097/QAD.0b013e32833243e7

[13] Gray ES, Madiga MC, Hermanus T, Moore PL, Wibmer CK, et al (2011) The neutralization breadth of HIV-1 develops incrementally over four years and is associated with CD4+ T cell decline and high viral load during acute infection. J Virol 85: 4828–4840. 21389135 10.1128/JVI.00198-11 PMC3126191

[14] Blankson JN (2010) Control of HIV-1 replication in elite suppressors. Discov Med 9: 261–266. 20350494

[15] Doria-Rose NA, Klein RM, Manion MM, O'Dell S, Phogat A, et al (2009) Frequency and phenotype of human immunodeficiency virus envelope-specific B cells from patients with broadly cross-neutralizing antibodies. J Virol 83: 188–199. 18922865 10.1128/JVI.01583-08 PMC2612342

[16] Scheid JF, Mouquet H, Feldhahn N, Seaman MS, Velinzon K, et al (2009) Broad diversity of neutralizing antibodies isolated from memory B cells in HIV-infected individuals. Nature 458: 636–640. 19287373 10.1038/nature07930

[17] Falkensammer B, Freissmuth D, Hubner L, Speth C, Dierich MP, et al (2007) Changes in HIV-specific antibody responses and neutralization titers in patients under ART. Front Biosci 12: 2148–2158. 17127452 10.2741/2218

[18] Binley JM, Trkola A, Ketas T, Schiller D, Clas B, et al (2000) The effect of highly active antiretroviral therapy on binding and neutralizing antibody responses to human immunodeficiency virus type 1 infection. J Infect Dis 182: 945–949. 10950795 10.1086/315774

[19] Markowitz M, Vesanen M, Tenner-Racz K, Cao Y, Binley JM, et al (1999) The effect of commencing combination antiretroviral therapy soon after human immunodeficiency virus type 1 infection on viral replication and antiviral immune responses. J Infect Dis 179: 527–537. 9952358 10.1086/314628

[20] Medina-Ramirez M, Sanchez-Merino V, Sanchez-Palomino S, Merino-Mansilla A, Ferreira CB, et al (2011) Broadly cross-neutralizing antibodies in HIV-1 patients with undetectable viremia. J Virol 85: 5804–5813. 21471239 10.1128/JVI.02482-10 PMC3126317

[21] Moir S, Buckner CM, Ho J, Wang W, Chen J, et al (2010) B cells in early and chronic HIV infection: evidence for preservation of immune function associated with early initiation of antiretroviral therapy. Blood 116: 5571–5579. 20837780 10.1182/blood-2010-05-285528 PMC3031405

[22] Regidor DL, Detels R, Breen EC, Widney DP, Jacobson LP, et al (2011) Effect of highly active antiretroviral therapy on biomarkers of B-lymphocyte activation and inflammation. AIDS 25: 303–314. 21192231 10.1097/QAD.0b013e32834273ad PMC3322644

[23] Bailey JR, Lassen KG, Yang HC, Quinn TC, Ray SC, et al (2006) Neutralizing antibodies do not mediate suppression of human immunodeficiency virus type 1 in elite suppressors or selection of plasma virus variants in patients on highly active antiretroviral therapy. J Virol 80: 4758–4770. 16641269 10.1128/JVI.80.10.4758-4770.2006 PMC1472047

[24] Saksena NK, Wang B, Zhou L, Soedjono M, Ho YS, et al (2010) HIV reservoirs in vivo and new strategies for possible eradication of HIV from the reservoir sites. HIV AIDS (Auckl) 2: 103–122. 22096389 10.2147/hiv.s6882 PMC3218690

[25] Massanella M, Martinez-Picado J, Blanco J (2013) Attacking the HIV reservoir from the immune and viral perspective. Curr HIV/AIDS Rep 10: 33–41. 23242702 10.1007/s11904-012-0150-8

[26] Ng CT, Jaworski JP, Jayaraman P, Sutton WF, Delio P, et al (2010) Passive neutralizing antibody controls SHIV viremia and enhances B cell responses in infant macaques. Nature Med 16: 1117–1119. 20890292 10.1038/nm.2233 PMC2952052

[27] Hessell AJ, Poignard P, Hunter M, Hangartner L, Tehrani DM, et al (2009) Effective, low-titer antibody protection against low-dose repeated mucosal SHIV challenge in macaques. Nature Med 15: 951–954. 19525965 10.1038/nm.1974 PMC4334439

[28] Hessell AJ, Rakasz EG, Poignard P, Hangartner L, Landucci G, et al (2009) Broadly neutralizing human anti-HIV antibody 2G12 is effective in protection against mucosal SHIV challenge even at low serum neutralizing titers. PLoS Pathog 5: e1000433. 19436712 10.1371/journal.ppat.1000433 PMC2674935

[29] Parren PW, Marx PA, Hessell AJ, Luckay A, Harouse J, et al (2001) Antibody protects macaques against vaginal challenge with a pathogenic R5 simian/human immunodeficiency virus at serum levels giving complete neutralization in vitro. J Virol 75: 8340–8347. 11483779 10.1128/JVI.75.17.8340-8347.2001 PMC115078

[30] Mascola JR, Stiegler G, VanCott TC, Katinger H, Carpenter CB, et al (2000) Protection of macaques against vaginal transmission of a pathogenic HIV-1/SIV chimeric virus by passive infusion of neutralizing antibodies. Nature Med 6: 207–210. 10655111 10.1038/72318

[31] Mascola JR, Lewis MG, Stiegler G, Harris D, VanCott TC, et al (1999) Protection of Macaques against pathogenic simian/human immunodeficiency virus 89.6PD by passive transfer of neutralizing antibodies. J Virol 73: 4009–4018. 10196297 10.1128/jvi.73.5.4009-4018.1999 PMC104180

[32] Braibant M, Barin F (2013) The role of neutralizing antibodies in prevention of HIV-1 infection: what can we learn from the mother-to-child transmission context? Retrovirology 10: 103. 24099103 10.1186/1742-4690-10-103 PMC3851888

[33] Euler Z, van Gils MJ, Bunnik EM, Phung P, Schweighardt B, et al (2010) Cross-reactive neutralizing humoral immunity does not protect from HIV type 1 disease progression. J Infect Dis 201: 1045–1053. 20170371 10.1086/651144

[34] Humbert M, Dietrich U (2006) The role of neutralizing antibodies in HIV infection. AIDS Rev 8: 51–59. 16848273

[35] Nelson JD, Brunel FM, Jensen R, Crooks ET, Cardoso RM, et al (2007) An affinity-enhanced neutralizing antibody against the membrane-proximal external region of human immunodeficiency virus type 1 gp41 recognizes an epitope between those of 2F5 and 4E10. J Virol 81: 4033–4043. 17287272 10.1128/JVI.02588-06 PMC1866125

[36] Burton DR, Pyati J, Koduri R, Sharp SJ, Thornton GB, et al (1994) Efficient neutralization of primary isolates of HIV-1 by a recombinant human monoclonal antibody. Science 266: 1024–1027. 7973652 10.1126/science.7973652

[37] Buchacher A, Predl R, Strutzenberger K, Steinfellner W, Trkola A, et al (1994) Generation of human monoclonal antibodies against HIV-1 proteins; electrofusion and Epstein-Barr virus transformation for peripheral blood lymphocyte immortalization. AIDS Res Hum Retroviruses 10: 359–369. 7520721 10.1089/aid.1994.10.359

[38] Thali M, Moore JP, Furman C, Charles M, Ho DD, et al (1993) Characterization of conserved human immunodeficiency virus type 1 gp120 neutralization epitopes exposed upon gp120-CD4 binding. J Virol 67: 3978–3988. 7685405 10.1128/jvi.67.7.3978-3988.1993 PMC237765

[39] Gray ES, Moore PL, Choge IA, Decker JM, Bibollet-Ruche F, et al (2007) Neutralizing antibody responses in acute human immunodeficiency virus type 1 subtype C infection. J Virol 81: 6187–6196. 17409164 10.1128/JVI.00239-07 PMC1900112

[40] Ingale S, Gach JS, Zwick MB, Dawson PE (2010) Synthesis and analysis of the membrane proximal external region epitopes of HIV-1. J Pept Sci 16: 716–722. 21104968 10.1002/psc.1325

[41] Nakamura KJ, Gach JS, Jones L, Semrau K, Walter J, et al (2010) 4E10-resistant HIV-1 isolated from four subjects with rare membrane-proximal external region polymorphisms. PLoS One 5: e9786. 20352106 10.1371/journal.pone.0009786 PMC2843716

[42] Brunel FM, Zwick MB, Cardoso RM, Nelson JD, Wilson IA, et al (2006) Structure-function analysis of the epitope for 4E10, a broadly neutralizing human immunodeficiency virus type 1 antibody. J Virol 80: 1680–1687. 16439525 10.1128/JVI.80.4.1680-1687.2006 PMC1367132

[43] Gach JS, Quendler H, Tong T, Narayan KM, Du SX, et al (2013) A Human Antibody to the CD4 Binding Site of gp120 Capable of Highly Potent but Sporadic Cross Clade Neutralization of Primary HIV-1. PLoS One 8: e72054. 23991039 10.1371/journal.pone.0072054 PMC3753353

[44] Gach JS, Furtmuller PG, Quendler H, Messner P, Wagner R, et al (2010) Proline is not uniquely capable of providing the pivot point for domain swapping in 2G12, a broadly neutralizing antibody against HIV-1. J Biol Chem 285: 1122–1127. 19903812 10.1074/jbc.M109.058792 PMC2801240

[45] Walker LM, Simek MD, Priddy F, Gach JS, Wagner D, et al (2010) A limited number of antibody specificities mediate broad and potent serum neutralization in selected HIV-1 infected individuals. PLoS Pathog 6: e1001028. 20700449 10.1371/journal.ppat.1001028 PMC2916884

[46] Ackerman ME, Moldt B, Wyatt RT, Dugast AS, McAndrew E, et al (2011) A robust, high-throughput assay to determine the phagocytic activity of clinical antibody samples. J Immunol Methods 366: 8–19. 21192942 10.1016/j.jim.2010.12.016 PMC3050993

[47] Avettand-Fenoel V, Chaix ML, Blanche S, Burgard M, Floch C, et al (2009) LTR real-time PCR for HIV-1 DNA quantitation in blood cells for early diagnosis in infants born to seropositive mothers treated in HAART area (ANRS CO 01). J Med Virol 81: 217–223. 19107966 10.1002/jmv.21390

[48] Ruprecht CR, Krarup A, Reynell L, Mann AM, Brandenberg OF, et al (2011) MPER-specific antibodies induce gp120 shedding and irreversibly neutralize HIV-1. J Exp Med 208: 439–454. 21357743 10.1084/jem.20101907 PMC3058584

[49] Kwong PD, Mascola JR (2012) Human antibodies that neutralize HIV-1: identification, structures, and B cell ontogenies. Immunity 37: 412–425. 22999947 10.1016/j.immuni.2012.08.012 PMC4706166

[50] Stamatatos L, Morris L, Burton DR, Mascola JR (2009) Neutralizing antibodies generated during natural HIV-1 infection: good news for an HIV-1 vaccine? Nat Med 15: 866–870. 19525964 10.1038/nm.1949

[51] Killian MS, Norris PJ, Rawal BD, Lebedeva M, Hecht FM, et al (2006) The effects of early antiretroviral therapy and its discontinuation on the HIV-specific antibody response. AIDS Res Hum Retroviruses 22: 640–647. 16831088 10.1089/aid.2006.22.640

[52] Morris L, Binley JM, Clas BA, Bonhoeffer S, Astill TP, et al (1998) HIV-1 antigen-specific and -nonspecific B cell responses are sensitive to combination antiretroviral therapy. J Exp Med 188: 233–245. 9670036 10.1084/jem.188.2.233 PMC2212446

[53] Kim JH, Mascola JR, Ratto-Kim S, VanCott TC, Loomis-Price L, et al (2001) Selective increases in HIV-specific neutralizing antibody and partial reconstitution of cellular immune responses during prolonged, successful drug therapy of HIV infection. AIDS Res Hum Retroviruses 17: 1021–1034. 11485619 10.1089/088922201300343708

[54] Haynes BF, Kelsoe G, Harrison SC, Kepler TB (2012) B-cell-lineage immunogen design in vaccine development with HIV-1 as a case study. Nat Biotechnol 30: 423–433. 22565972 10.1038/nbt.2197 PMC3512202

[55] Liao HX, Chen X, Munshaw S, Zhang R, Marshall DJ, et al (2011) Initial antibodies binding to HIV-1 gp41 in acutely infected subjects are polyreactive and highly mutated. J Exp Med 208: 2237–2249. 21987658 10.1084/jem.20110363 PMC3201211

[56] Huang J, Ofek G, Laub L, Louder MK, Doria-Rose NA, et al (2012) Broad and potent neutralization of HIV-1 by a gp41-specific human antibody. Nature 491: 406–412. 23151583 10.1038/nature11544 PMC4854285

[57] Pejchal R, Gach JS, Brunel FM, Cardoso RM, Stanfield RL, et al (2009) A conformational switch in human immunodeficiency virus gp41 revealed by the structures of overlapping epitopes recognized by neutralizing antibodies. J Virol 83: 8451–8462. 19515770 10.1128/JVI.00685-09 PMC2738203

[58] Zwick MB, Jensen R, Church S, Wang M, Stiegler G, et al (2005) Anti-human immunodeficiency virus type 1 (HIV-1) antibodies 2F5 and 4E10 require surprisingly few crucial residues in the membrane-proximal external region of glycoprotein gp41 to neutralize HIV-1. J Virol 79: 1252–1261. 15613352 10.1128/JVI.79.2.1252-1261.2005 PMC538539

[59] Song L, Sun ZY, Coleman KE, Zwick MB, Gach JS, et al (2009) Broadly neutralizing anti-HIV-1 antibodies disrupt a hinge-related function of gp41 at the membrane interface. Proceedings of the National Academy of Sciences of the United States of America 106: 9057–9062. 19458040 10.1073/pnas.0901474106 PMC2690059

[60] Zwick MB (2005) The membrane-proximal external region of HIV-1 gp41: a vaccine target worth exploring. AIDS 19: 1725–1737. 16227780 10.1097/01.aids.0000189850.83322.41

[61] Gray ES, Madiga MC, Moore PL, Mlisana K, Abdool Karim SS, et al (2009) Broad neutralization of human immunodeficiency virus type 1 mediated by plasma antibodies against the gp41 membrane proximal external region. J Virol 83: 11265–11274. 19692477 10.1128/JVI.01359-09 PMC2772769

[62] Liu L, Cimbro R, Lusso P, Berger EA (2011) Intraprotomer masking of third variable loop (V3) epitopes by the first and second variable loops (V1V2) within the native HIV-1 envelope glycoprotein trimer. Proc Natl Acad Sci U S A 108: 20148–20153. 22128330 10.1073/pnas.1104840108 PMC3250183

[63] Rusert P, Krarup A, Magnus C, Brandenberg OF, Weber J, et al (2011) Interaction of the gp120 V1V2 loop with a neighboring gp120 unit shields the HIV envelope trimer against cross-neutralizing antibodies. J Exp Med 208: 1419–1433. 21646396 10.1084/jem.20110196 PMC3135368

[64] Gorny MK, Stamatatos L, Volsky B, Revesz K, Williams C, et al (2005) Identification of a new quaternary neutralizing epitope on human immunodeficiency virus type 1 virus particles. J Virol 79: 5232–5237. 15795308 10.1128/JVI.79.8.5232-5237.2005 PMC1069558

[65] Walker LM, Phogat SK, Chan-Hui PY, Wagner D, Phung P, et al (2009) Broad and potent neutralizing antibodies from an African donor reveal a new HIV-1 vaccine target. Science 326: 285–289. 19729618 10.1126/science.1178746 PMC3335270

[66] Zolla-Pazner S, Cardozo T (2010) Structure-function relationships of HIV-1 envelope sequence-variable regions refocus vaccine design. Nature reviews Immunology 10: 527–535. 10.1038/nri2801 PMC3167078 20577269

[67] Frost SD, Wrin T, Smith DM, Kosakovsky Pond SL, Liu Y, et al (2005) Neutralizing antibody responses drive the evolution of human immunodeficiency virus type 1 envelope during recent HIV infection. Proc Natl Acad Sci U S A 102: 18514–18519. 16339909 10.1073/pnas.0504658102 PMC1310509

[68] Rong R, Bibollet-Ruche F, Mulenga J, Allen S, Blackwell JL, et al (2007) Role of V1V2 and other human immunodeficiency virus type 1 envelope domains in resistance to autologous neutralization during clade C infection. J Virol 81: 1350–1359. 17079307 10.1128/JVI.01839-06 PMC1797511

[69] Davis KL, Gray ES, Moore PL, Decker JM, Salomon A, et al (2009) High titer HIV-1 V3-specific antibodies with broad reactivity but low neutralizing potency in acute infection and following vaccination. Virology 387: 414–426. 19298995 10.1016/j.virol.2009.02.022 PMC2792036

[70] Pancera M, Shahzad-Ul-Hussan S, Doria-Rose NA, McLellan JS, Bailer RT, et al (2013) Structural basis for diverse N-glycan recognition by HIV-1-neutralizing V1-V2-directed antibody PG16. Nat Struct Mol Biol 20: 804–813. 23708607 10.1038/nsmb.2600 PMC4046252

[71] Pejchal R, Doores KJ, Walker LM, Khayat R, Huang PS, et al (2011) A potent and broad neutralizing antibody recognizes and penetrates the HIV glycan shield. Science 334: 1097–1103. 21998254 10.1126/science.1213256 PMC3280215

[72] Forthal DN, Moog C (2009) Fc receptor-mediated antiviral antibodies. Curr Opin HIV AIDS 4: 388–393. 20048702 10.1097/COH.0b013e32832f0a89 PMC2882066

[73] Holl V, Peressin M, Moog C (2009) Antibody-Mediated Fcgamma Receptor-Based Mechanisms of HIV Inhibition: Recent Findings and New Vaccination Strategies. Viruses 1: 1265–1294. 21994593 10.3390/v1031265 PMC3185537

