# Supplementary figures and images for: HIV-1 Specific Antibody Titers and Neutralization among Chronically Infected Patients on Long-Term Suppressive Antiretroviral Therapy (ART): A Cross-Sectional Study
Source: PLoS One. 2014 Jan 15;9(1):e85371. doi: 10.1371/journal.pone.0085371 (PMC3893210; doi:10.1371/journal.pone.0085371)

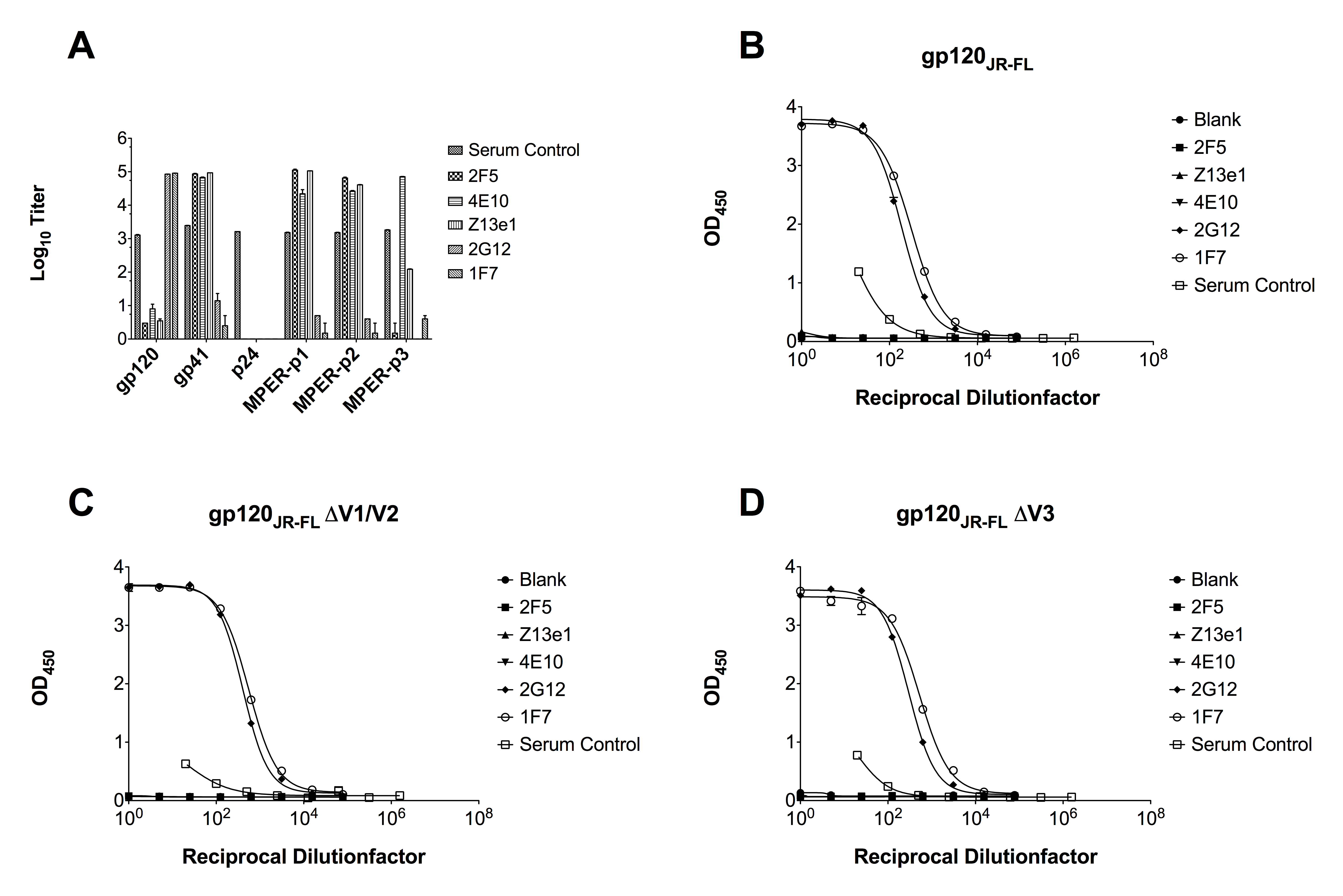

Supplement: Figure S1 — Monoclonal antibody and serum control binding titers against various antigens. (A), gp120JR-FL wild type (B), gp120JR-FL V1/V2 loop deletion variant C), and gp120JR-FL V3 loop deletion variant (D). 2G12 and 1F7 only recognized gp120JR-FL, whereas 2F5, Z13e1, and 4E10 interacted with gp41 and MPER. MPER-p2 was only recognized by mAb 4E10 since the epitopes of 2F5 and Z13e1 are missing No significant binding was detected against p24. The human serum control revealed only minor binding against all tested antigens. (TIF) [file pone.0085371.s001.tif]
