# Supplementary material for: HIV-1 Specific Antibody Titers and Neutralization among Chronically Infected Patients on Long-Term Suppressive Antiretroviral Therapy (ART): A Cross-Sectional Study
Source: PLoS One. 2014 Jan 15;9(1):e85371. doi: 10.1371/journal.pone.0085371 (PMC3893210; doi:10.1371/journal.pone.0085371)
Supplement: Table S1 — EC50 values of three potential CD4 binding site antibody- containing serum samples. (DOCX) [file pone.0085371.s002.docx]

**Table S1.** EC_50_ values of three potential CD4 binding site antibody- containing serum samples.

| Sample | EC_50_^*^ | |
| --- | --- | --- |
|  | gp120_JR-FL_ | gp120_JR-FL_ + sCD4 |
| #12 | 3828 | 5689 |
| #37 | 3358 | 3725 |
| #42 | 4280 | 5645 |

^*^ Reciprocal serum dilution factor
