# Supplementary material for: HIV-1 Specific Antibody Titers and Neutralization among Chronically Infected Patients on Long-Term Suppressive Antiretroviral Therapy (ART): A Cross-Sectional Study
Source: PLoS One. 2014 Jan 15;9(1):e85371. doi: 10.1371/journal.pone.0085371 (PMC3893210; doi:10.1371/journal.pone.0085371)
Supplement: Table S2 — Neutralization assay with HIV-1JR-FL and HIV-2/HIV-1 MPER chimeras using monoclonal antibody controls. (DOCX) [file pone.0085371.s003.docx]

**Table S2.** Neutralization assay with HIV-1_JR-FL_ and HIV-2/HIV-1 MPER chimeras using monoclonal antibody controls.

| MAbs | IC_50_ (μg/mL) | | | | |
| --- | --- | --- | --- | --- | --- |
|  | Virus | | | | |
|  | JR-FL | VSV-g | 7312-C1 | 7312-C1C | 7312A |
| 1F7 | 0.01 | > 5 | > 5 | > 5 | > 5 |
| 2G12 | 1.0 | > 5 | > 5 | > 5 | > 5 |
| 2F5 | 1.5 | > 5 | < 0.2 | > 5 | > 5 |
| 4E10 | > 5 | > 5 | < 0.2 | < 0.2 | > 5 |

Note: Data represent the means of two independent experiments, each done in duplicate.
